# Detecting and Profiling Endogenous RNA G-Quadruplexes in the Human Transcriptome

**DOI:** 10.3390/ijms22158012

**Published:** 2021-07-27

**Authors:** Rongxin Zhang, Yajun Liu, Xingxing Zhang, Ke Xiao, Yue Hou, Hongde Liu, Xiao Sun

**Affiliations:** 1 State Key Laboratory of Bioelectronics, School of Biological Science and Medical Engineering, Southeast University, Nanjing 210096, China; rongxinzhang@outlook.com (R.Z.); liuyajun_biology@126.com (Y.L.); zhangxingxing114@foxmail.com (X.Z.); kexiao@seu.edu.cn (K.X.); houyue@xjtu.edu.cn (Y.H.); liuhongde@seu.edu.cn (H.L.); 2 Hangzhou Hongquan Internet of Things Technology Co., Ltd., Hangzhou 310012, China; 3 Key Laboratory of Biomedical Information Engineering of Ministry of Education, School of Life Science and Technology, Xi’an Jiaotong University, Xi’an 710049, China

**Keywords:** G-quadruplex, transcriptome, post-transcriptional regulation, miRNA targeting, RG4, RNA G-quadruplex

## Abstract

G-quadruplexes are the non-canonical nucleic acid structures that are preferentially formed in G-rich regions. This structure has been shown to be associated with many biological functions. Regardless of the broad efforts on DNA G-quadruplexes, we still have limited knowledge on RNA G-quadruplexes, especially in a transcriptome-wide manner. Herein, by integrating the DMS-seq and the bioinformatics pipeline, we profiled and depicted the RNA G-quadruplexes in the human transcriptome. The genes that contain RNA G-quadruplexes in their specific regions are significantly related to immune pathways and the COVID-19-related gene sets. Bioinformatics analysis reveals the potential regulatory functions of G-quadruplexes on miRNA targeting at the scale of the whole transcriptome. In addition, the G-quadruplexes are depleted in the putative, not the real, PAS-strong poly(A) sites, which may weaken the possibility of such sites being the real cleaved sites. In brief, our study provides insight into the potential function of RNA G-quadruplexes in post-transcription.

## 1. Introduction

G-quadruplexes are the non-canonical nucleic acid secondary structures that are formed by stacking G-quartets on top of each other. Each G-quartet planar comprises four guanines, where adjacent guanines are bonded to each other via Hoogsteen hydrogen [1]. The stability of G-quadruplexes is partially determined by monovalent cations which are located within or between the plane of G-quartets. G-quadruplexes can adopt parallel, antiparallel or hybrid structures, which mainly depend on the orientation of the strands. The number and orientation of the strands highlight the diversity of G-quadruplex structure topologies [1]. Computational methods have identified more than 350,000 DNA G-quadruplex-forming sequences in the human genome, which are usually located in some important genomic regions, such as promoters and telomeres, indicating that G-quadruplexes are involved in many critical biological processes [2].

DNA G-quadruplexes have been extensively studied in the past few decades. In recent years, researchers started to focus on RNA G-quadruplexes. In 2007, Sunita Kumari et al. demonstrated the RNA G-quadruplex in the 5′-UTR (untranslated region) of NRAS (neuroblastoma RAS viral oncogene homolog) proto-oncogene repress translation [3]. After that, a considerable amount of research was carried out at the single transcription level with the aim of understanding the relationship between RNA G-quadruplexes in 5′-UTR and translation [4,5,6,7]. Another report revealed that the RNA G-quadruplexes can act as a translational repressor only when they are located near the 5′ cap of NRAS [8]. However, the above studies were mainly based on biological experiments in the scope of single RNA, and insights at the transcriptome-wide scale are still insufficient. A systematic study revealed that the putative RNA G-quadruplexes (G_x_N_1-7_G_x_N_1-7_G_x_N_1-7_G_x_, where x ≥ 3 and N can be any base) in UTRs were under selective constraints and were tightly associated with several biological functions [9]. Individual studies showed that G-quadruplexes existed in the human transcriptome in vitro [10] and the Arabidopsis transcriptome [11]. Nevertheless, whether RNA G-quadruplexes are folded in the normal physiological state of the cells remains to be elucidated and studied in detail.

Moreover, the SARS-unique domain (SUD) was confirmed to preferentially bind to the RNA G-quadruplex on the host mRNAs [12], and a similar domain was retained in SARS-CoV-2 [13], which is potentially related to the interference mechanism of SARS-CoV-2. Therefore, it is critical to characterize and interpret the RNA G-quadruplexes at the whole-transcriptome scale.

DMS-seq is one of the most robust methods that can be used to detect the RNA secondary structures [14]. Dimethyl sulfate (DMS) can methylate the adenines and cytosines that are not in the Watson–Crick pairing or other forms of base pairing [14,15]. The methylated bases could be detected as they stall reverse transcription one nucleotide before the modified bases, and the base-pairing status of other bases, including guanines and thymines, could be evaluated from the sequencing data [14,15]. In this study, we employed DMS-based structure-seq coupled with computational analysis to detect RNA G-quadruplexes in the YanHuang cell line, which is a Chinese population cell line. Most of the RNA G-quadruplex-related studies are mainly based on cancer cell lines, and therefore the conclusions respond to the properties of cancer cells, whereas we used non-cancer cell lines that can potentially reveal the function of RNA G-quadruplexes in a normal physiological state. Herein, we termed the RNA G-quadruplexes detected in the transcriptome as observed RNA G-quadruplexes (OG4s), while the RNA G-quadruplexes that are not folded were designated as Unfolded RNA G-quadruplexes (UG4s). The distribution mode of OG4s in the human transcriptome was depicted, and a notable non-uniform distribution feature was observed. Additionally, the pathway enrichment analysis was performed for the OG4-associated gene clusters. We also investigated the potential relationship between OG4s and miRNA binding sites. In addition, we evaluated the distribution pattern of OG4s to the putative, not the genuine, PAS-strong (Polyadenylation Signal) poly(A) sites. In general, our analysis indicated that the OG4s are correlated with post-transcriptional events, which emphasizes the importance of OG4s in the regulation of biological processes. To our knowledge, this is the first study to depict the whole-transcriptome RNA G-quadruplexes in the cell line of the Chinese population, and our study supports the formation of RNA G-quadruplexes in the human transcriptome.

## 2. Results

### 2.1. Profiling of the Observed RNA G-Quadruplexes

**Figure 1:**
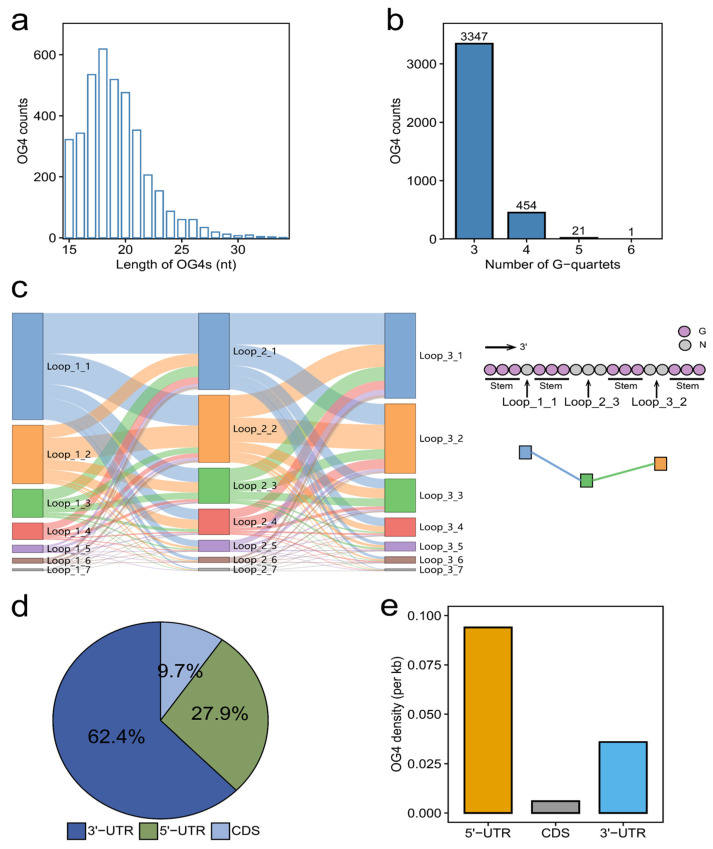
Characterization of OG4s in the transcriptome of the YanHuang cell line. (**a**,**b**) The counts of OG4s with different lengths and numbers of G-quartets. (**c**) The loop composition of OG4s. The Sankey diagram of the loop length composition for each OG4 is displayed in the left panel. The label Loop_*X*_*Y* is interpreted as follows: the *X* ($X\in \;\left\{1,2,3\right\}$) represents the three loops in OG4s, while the *Y* standards for the length of a loop ($Y\in \;\left\{1,2,3,4,5,6,7\right\}$). There is an example in the right panel. In this case, The OG4 contains three loops, the first of which is of 1 nt, namely Loop_1_1; likewise, the second and third loops are encoded as Loop_2_3 and Loop_3_2 according to their lengths, respectively. (**d**,**e**) The proportion and density of OG4s in different regions of mRNAs.

In this study, we focused on the canonical RNA G-quadruplexes (G_x_N_1−7_G_x_N_1−7_G_x_N_1−7_G_x_, where x ≥ 3 and N can be any base). The length of OG4s exhibits a left-skewed distribution with an interval between 15 and 34 nucleotides (nt), and most of the OG4 lengths are shorter than 24 nt (Figure 1a). G-quartets are tightly associated with the thermal stability of G-quadruplexes. We found that the overwhelming majority of OG4s are made up of three G-quartet layers (Figure 1b). In addition to the number of G-quartets, the size of the three loops located in the OG4s can also affect their thermal stability. Previous studies have revealed the negative correlation between the loop length and the thermal stability of G-quadruplexes [16,17]. In line with the previous reports, our study indicated that the OG4s with shorter loop lengths account for a larger proportion (Figure 1c), which implies their high thermal stability under physiological conditions when compared to the OG4s with longer loop sizes. To be specific, the OG4s with a total loop length of less than 10 nt occupy approximately 90% of all the OG4s. More than half (57.1%) of the OG4s have all three loops with lengths of no more than three nt. We further annotated the positions of OG4s on their corresponding mRNAs and counted the number of OG4s in different mRNA regions (5′-UTR, CDS, and 3′-UTR). The counts of OG4s present significant differences in the 5′-UTR, CDS, and 3′-UTR (Figure 1d). Nearly three fifths of the OG4s were observed as being located in the 3′-UTR (62.4%), while the proportions of OG4s in the 5′-UTR (27.9%) and CDS (9.7%) are notably less than those in the 3′-UTR. Considering that the average lengths of the 5′-UTR, CDS, and 3′-UTR are quite different, we subsequently calculated the density of OG4s in these regions (Figure 1e). Interestingly, there exists a discrepancy between the counts and the density of OG4s in different regions of mRNAs. Although a multitude of OG4s were located in the 3′-UTR, when observing the density of OG4s, the 5′-UTR was the densest region of OG4s, while the CDSs had the lowest density of OG4s.

### 2.2. Pathway Analysis of the UTR-OG4-Containing Genes

It is acknowledged that the G-quadruplexes in UTRs are functional and may have roles in the regulation of biological processes. To determine the pathways associated with the gene sets containing OG4s in UTRs, we performed a pathway analysis based on the occupancy pattern of OG4s.

**Figure 2:**
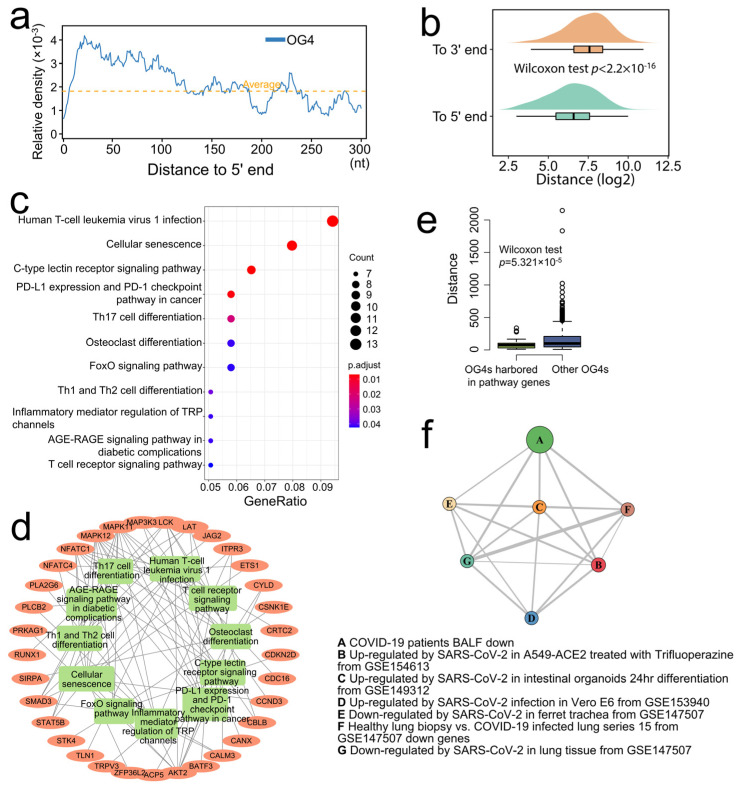
Pathway analysis of the OG4 related genes. (**a**) The abscissa represents the distance to the 5′ end, as the ordinate indicates the relative OG4 density value. The relative OG4 density signal is shown as a blue polyline, while the average OG4 density in the whole 5′-UTR is marked by an orange dotted line. (**b**) The raincloud plot shows the distance of OG4s to the 5′ and 3′ ends of the 5′-UTRs, respectively. (**c**) The dot plot shows the result of the KEGG pathway over-representation test. The abscissa and ordinate designate the gene ratio in a specific pathway and the enriched pathways. The dot size and dot color display the count of genes in a pathway and the FDR *p*-value, respectively. (**d**) The gene conception network of the enriched pathways. The inner circle implies the significant pathways that are marked with green rectangles, while the genes are signified by the orange oval nodes. A gene node would be linked to a pathway node if the gene were involved in the pathway. (**e**) The boxplot shows the distance of OG4s to the 5′ end of the 5′-UTRs. The left (green) box indicates the OG4s that are located in the 5′-UTR of enriched genes, while the right (blue) box indicates the OG4s that are located in the 5′-UTR of other genes. (**f**) The network graph illustrates the connection (overlapped genes) of the highly significantly enriched COVID-19-related gene sets. A to G, respectively, represent the seven COVID-19-related gene sets. The node size denotes the relatively adjusted *p*-value for each gene set. The relative overlapped gene number between two nodes is marked as the thickness of the edges.

The former findings provide a hint that only when the RNA G-quadruplexes are close enough to the 5′ end can they act as translational repressors [8]. The relative density of OG4s within the first 300 nt of the 5′-UTRs in all mRNAs was first calculated. In addition, the average density of OG4s in all the 5′-UTRs was computed as the background signal. Significant enrichment of OG4s in the first 100 nt of the 5′-UTRs was observed with a peak at about position 25 nt, and no enrichment was identified after 100 nt compared with the background signal (Figure 2a). Compared to the 3′ end of the 5′-UTRs, the OG4s located at the 5′-UTRs were closer to the 5′ end (Figure 2b, one-sided Wilcoxon test, *p* < 2.2 × 10^−16^).

Then, we collected the genes with one or more OG4s present in the first 100 nt of their 5′-UTRs. The KEGG (Kyoto Encyclopedia of Genes and Genomes) over-representation test was performed to see which biological pathways are possibly affected by the gene set. As a result, several pathways were significantly enriched (FDR < 0.05), such as the pathways that are related to the differentiation of Th17 (T-helper 17), Th1 (T-helper 1), and Th2 (T-helper 2) cells and the pathway involving in the regulation of PD1/PD-L1 (Programmed cell Death protein 1/Programmed Death-Ligand 1), which were closely related to the immune balance and events (Figure 2c, Supplementary Table S1). We next observed the enriched genes that are involved in the significant pathways. Approximately half of the genes were presented in multiple pathways (Figure 2d), which emphasized its importance in regulating such pathways. In addition, the OG4s harbored in the 5′-UTR of enriched genes were closer to the 5′ end of 5′-UTRs as compared with other 5′-UTR-OG4s (Figure 2e, one-sided Wilcoxon test, *p* = 5.321 × 10^−5^), which is conducive to the regulation of translation when considering the distance effect [8]. The above results highlight the potential role of RNA G-quadruplexes in the dynamic regulation of immune responses at the scale of the whole transcriptome.

A previous study indicated that the SARS-Unique Domain (SUD) in SARS-CoV preferentially binds to the G-quadruplex structures in the 3′-UTR of the host transcripts and may be responsible for its high pathogenicity by obstructing the stability of the host transcripts [12]. In addition, we have reported that there exists a SUD-homology structure in SARS-CoV-2, which implies that SARS-CoV-2 may retain similar interference capability [13]. The Enrichr database has collected many datasets related to COVID-19 from various studies, which embodies hundreds of differential expression signature gene sets due to SARS-CoV-2 infection. For instance, the dysregulated (up- and downregulated) gene sets that were derived from the SARS-CoV-2-infected lung tissues and the SARS-CoV-2-infected cell lines with different multiplicities of infection (MOIs), including A549, Calu-3, etc. Thus, another over-representation test was conducted in the Enrichr online service to explore whether the genes containing G-quadruplexes in its 3′-UTR are associated with COVID-19-related gene sets. Surprisingly, many gene sets were significantly enriched, including the sets of differentially (both up- and downregulated) expressed genes in intestinal organoids that were infected by SARS-CoV-2 and many other COVID-19-related gene sets. (Benjamini–Hochberg *p* < 0.05, Supplementary Table S2). The gene conception network of the gene sets was visualized with a more stringent cutoff (Figure 2f, Benjamini–Hochberg *p* < 0.0001). Those COVID-19-related gene sets were closely relevant to the genes possessing OG4s in its 3′-UTR, which leads us to speculate that SARS-CoV-2 may interfere with the gene expression of some important genes through binding to the G-quadruplex structures in host transcripts; however, direct evidence is needed to clarify the interference mechanism of SARS-CoV-2.

### 2.3. RNA G-Quadruplexes Are Associated with miRNA Target Sites

Previous studies have suggested that the mRNA secondary structures and their stability may affect the targeting of miRNAs [18]. Therefore, we started to explore the potential relationship between OG4s and miRNA target sites on a transcriptome-wide scale.

**Figure 3:**
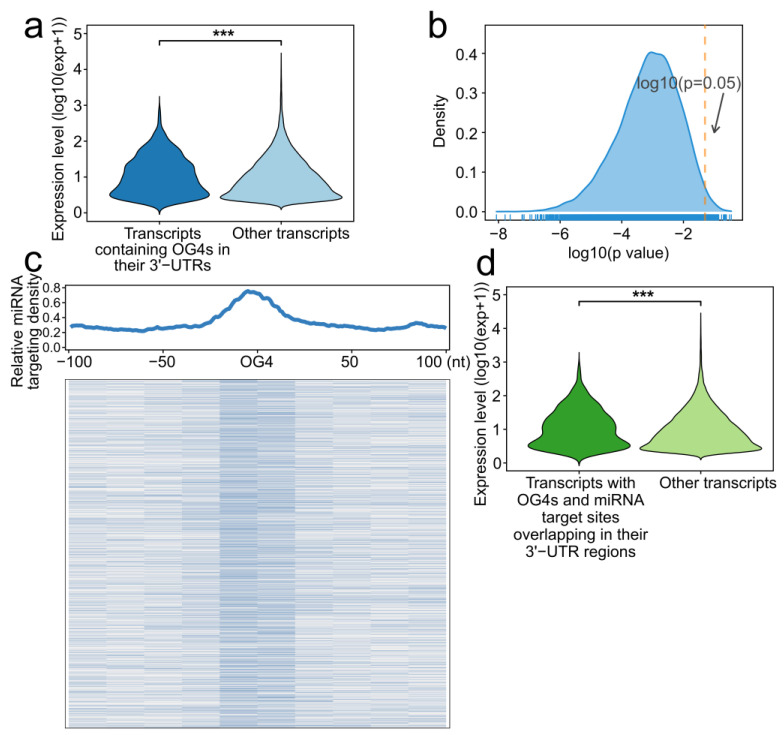
RNA G-quadruplexes are related to miRNA binding. (**a**) Violin plot showing the expression level of the transcripts containing OG4s in their 3′-UTRs and other transcripts (***, *p*-value < 0.001). (**b**) Density plot of the *p*-value for all simple random sampling rounds. The orange dashed line corresponds to the *p*-value equal to 0.05. (**c**) Top panel: Profile of miRNA target density around all OG4s. The abscissa indicates the distance of 100 nt upstream and downstream of the OG4s, while the abscissa indicates the relative density of the miRNA target sites. Bottom panel: Heatmap of the miRNA target sites density around all OG4s with a resolution of 20 nt. (**d**) Violin plot of the expression level for the transcripts with OG4s and miRNA target sites overlapping in their 3′-UTRs and other transcripts (***, *p*-value < 0.001).

We first divided all the transcripts into two groups based on whether the 3′-UTR of the transcript contained OG4s. The expression level difference between the two groups was then assessed. As a result, the expression level of the transcripts that contained OG4 in their 3′-UTR was significantly higher than those without OG4s in their 3′-UTRs (Figure 3a, one-sided Wilcoxon test, *p* = 9.063 × 10^−6^). Considering that the transcripts without OG4s in their 3′-UTRs were heavily outnumbered by the 3′-UTR-OG4-containing transcripts, we conducted a simple random sampling of the mRNAs 10,000 times (Please see Methods). We visualized the distribution of all *p*-values obtained from sampling (Figure 3b). Nearly all the rounds (9,833/10,000) showed that the expression of 3′-UTR-OG4 containing transcripts was significantly greater than the random transcript set of equal size.

We next set out to determine the distribution of miRNA target sites around OG4 sequences. All the human high confidence miRNA sequences from the miRBase database were collected, and 897 miRNAs were eventually obtained. MiRmap and miRanda were performed to predict the miRNA target sites, and only identical prediction results were employed as putative miRNA target sites. Moreover, an equal number of randomly selected regions were also generated as a contrast to the OG4 sequences. Intriguingly, we found that the miRNA target sites were enriched around OG4 sequences, while no enrichment was observed in randomly selected regions (Figure 3c, Supplementary Figure S1). The results suggest that OG4s tend to be located at miRNA target sites. In theory, the enrichment of miRNA target sites around OG4 sequences possibly reflects the specific target preference of miRNAs to OG4 sequences; however, it should be noted that those OG4 sequences are inclined to form RNA G-quadruplex secondary structures rather than linear sequences, which may impair the interaction between miRNAs and mRNAs in turn. MiRNAs exert their functions via degrading the target mRNAs or inhibiting translation, but the presence of the RNA G-quadruplex secondary structures on the target sites could block the binding of miRNAs, and thus restrain both regulatory mechanisms.

We further investigated the expression level of the transcripts with miRNA target sites and OG4s overlaps in their 3′-UTRs. These transcripts showed a higher expression level than other transcripts (Figure 3d, one-sided Wilcoxon test, *p* = 4.380872 × 10^−4^). Another simple random sampling was performed to eliminate the order-of-magnitude bias, and a considerable number of rounds (8690/10,000, Supplementary Figure S2) supported the hypothesis that the expression of such transcripts was significantly higher than the random transcripts.

Taken together, we assume that the RNA G-quadruplex might exist as a modulating element in 3′-UTR that could potentially regulate the targeting of miRNAs and thus protect the transcripts from degradation or inhibition.

### 2.4. RNA G-Quadruplexes Are Depleted from the Putative PAS-Strong Poly(A) Sites

**Figure 4:**
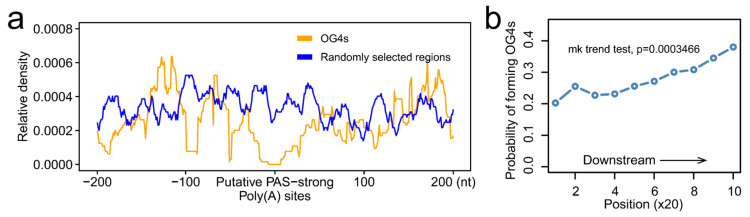
RNA G-quadruplexes are depleted in the vicinity of the putative PAS-strong poly(A) sites. (**a**) The distribution of OG4s in the window of 200 nt up and downstream of the putative PAS-strong poly(A) sites. The yellow line represents the relative density of OG4s, and the blue line represents the relative density of randomly selected regions. (**b**) The probability of forming OG4s downstream of the putative PAS-strong poly(A) sites at the resolution of 20 nt.

Accumulating evidence indicates that RNA G-quadruplexes play an important role in mRNA cleavage [19,20]. Therefore, we set out to address the question of whether RNA G-quadruplexes exhibit any distribution pattern downstream of the putative PAS-strong poly(A) sites. Putative PAS-strong poly(A) sites are the potential cleavage sites rather than real cleavage sites. The POLYAR program was executed to predict all the putative PAS-strong poly(A) sites in 3′-UTRs, and a total of 64,612 putative sites were predicted. Subsequently, we calculated the distribution of OG4s 200 nt upstream and downstream of the putative PAS-strong poly(A) sites. Intriguingly, a prominent depletion of OG4s was observed neighboring the putative PAS-strong poly(A) sites, as compared with that of the randomly selected regions (Figure 4a). Furthermore, when the distance to putative PAS-strong poly(A) sites exceeds 50 nt, the distribution of OG4s was similar to the randomly selected region signals. In addition, we visualized the distribution of UG4s around the putative PAS-strong poly(A) sites, and as a result, the analogous distribution was observed (Supplementary Figure S3). Taken together, for both OG4s and UG4s, there is a depletion phenomenon around the putative PAS-strong poly(A) sites. Because of the prior findings that the RNA G-quadruplexes downstream of the putative PAS-strong poly(A) sites may enhance the cleavage efficiency, we next investigated the probability of forming G4s downstream of the putative PAS-strong poly(A) sites at a resolution of 20 nt. Dramatically, we found that the probability of forming G4s had a distinct upward tendency (Figure 4b; Mann–Kendall trend test, *p* = 0.0003466, S = 41.0). This probability increased with the increase in the distance to the putative PAS-strong poly(A) sites, which allowed us to presume that the depletion of RNA G-quadruplexes in the vicinity of the putative PAS-strong poly(A) sites, especially the low probability of forming G4s, could potentially reduce the possibility of these putative sites being the real cleavage sites. In the absence of OG4s that are located directly downstream of the putative PAS-strong poly(A) sites, the effect of stalling the transcription complex may be weakened, eventually causing the transcriptional complex to pass over the putative PAS-strong poly(A) sites instead of being the real poly(A) sites.

## 3. Discussion

Despite the extensive research on DNA G-quadruplexes, the properties and functions of RNA G-quadruplexes still need to be elucidated. Recent progress in uncovering the role of RNA G-quadruplexes presents evidence for their relevance in post-transcriptional regulation. However, profiling the RNA G-quadruplexes at the scale of the whole transcriptome, particularly in human cells, is vital but insufficient. In this study, we applied the DMS-seq followed by the modified StructureFold software to probe the RNA G-quadruplexes in the YanHuang cell line. DMS-seq can provide nucleotide status, which can be used as experimental constraints to improve the prediction of RNA secondary structures. By adopting this strategy, we determined the OG4s on each transcript at single-nucleotide resolutions.

The OG4s discovered in the YanHuang cell line displayed shorter loops, which is in accordance with the physical chemistry experiment results showing that RNA G-quadruplexes with shorter loops usually possess higher thermal stability [16,17]. Compared with CDSs, UTRs contain a substantial part of the OG4s, and the density of OG4s that are harbored in UTRs is much higher than that of CDSs. Why OG4s are more concentrated in the UTRs rather than CDSs deserves further investigation. We suspected that the OG4s in CDSs may impede the elongation of ribosomes, hence impairing the translation, and this would be a “matter” for the translation process. To avoid this, the OG4s should be unfolded, which eventually leads to the low content and density of OG4s in the CDS.

We observed a notable enrichment of OG4s in the vicinity of the 5′ cap, and a previous study demonstrated that the RNA G-quadruplex, which is close enough to the 5′ cap, exhibits a repression effect in the NRAS proto-oncogene transcript, leading us to propose that these OG4s could act as modulation elements by impeding the assembly of translation initiation complexes. In addition, the relative density of OG4s decreased sharply when approaching the start codon (Supplementary Figure S4); however, no notable enrichment was found upstream of the start codon. Several reports pointed out that RNA G-quadruplex was a positive element for the IRES-mediated (Internal Ribosome Entry Site) translation, but no significant enrichment was observed upstream of the start codon in our study. In fact, the role of RNA G-quadruplexes in IRES-mediated translation is still controversial [21,22].

G-quadruplexes have been linked to numerous diseases due to their flexible and effective functions in regulating biological processes. We here show the suggestive indication that the RNA G-quadruplexes are tightly associated with immune events, such as T-cell differentiation. The immune-related cluster of genes, the 5′-UTRs which contain OG4s, may be dynamically controlled through the folding conformation of OG4s; this ultimately influences the immune response. Predictably, targeting the OG4s in immune-related genes might be a promising strategy for cancer treatment.

The mechanism of SARS-CoV-2 to disrupt the expression of host genes has attracted widespread attention, and several recent studies have discovered some potential mechanisms [23,24]. However, whether there are still some other potential interference mechanisms is a critical question, which could be influential for COVID-19 drug development. Notably, SARS-CoV-2 contains an amino acid sequence similar to that of SUD [13] which potentially binds to the G-quadruplex structures in the 3′-UTRs of host transcripts, leading to the disruption of host transcripts. The genes that contain OG4s in their 3′-UTRs are enriched in some COVID-19-related gene sets, such as the upregulated and downregulated gene sets in SARS-CoV-2-infected intestinal organoids and some other cell lines. We noticed that both the up-regulated and down-regulated sets were enriched; therefore, we speculate that, on the one hand, the SUD-homology structure may directly bind to the G-quadruplexes on host transcripts, leading to the changes in host transcripts’ stability; on the other hand, the SUD-homology structure may compete with microRNAs to bind host transcripts. Thus, investigating the role of the SUD-homology structure in dysregulating the expression of host transcripts via RNA G-quadruplex structures would be interesting. Notably, the binding of the SUD-homology structure to RNA G-quadruplexes can be simulated by molecular dynamics methods, as some progress has been made with this approach regarding SUD [25].

Our findings also yield suggestive evidence for the protective effect of RNA G-quadruplexes on transcripts, as they prevent the targeting of miRNAs. However, massive biological experiments should be undertaken to carefully confirm the impact of RNA G-quadruplexes on miRNA targeting in vivo. Particularly, it is necessary to investigate whether the RNA G-quadruplexes have dual effects on miRNA targeting. As Samuel Rouleau et al. described in their study [26], we here suggest again that RNA G-quadruplex structures should be considered as a dynamic regulation factor to improve the prediction of mRNA–miRNA interactions. Both the OG4s and UG4s are significantly depleted in the vicinity of putative PAS-strong poly(A) sites, and the probability of forming OG4s is increased with the increase in the distance from putative PAS-strong poly(A) sites. Combined with the prior reports, we hold that the RNA G-quadruplex structures are a positive signal for the polyadenylation events and may work as an enhancement element for the choosing of PAS-strong poly(A) sites. However, massive biological experiments are required to explore and demonstrate this relationship.

Increasing evidence has emphasized the important biological role of RNA G-quadruplexes and their close relationship with certain diseases [27,28], and we hope that more robust techniques can be developed to detect RNA G-quadruplexes with diverse complex structures [29] in vivo, or even at the single-cell level.

## 4. Materials and Methods

### 4.1. Cell Growth

We cultured the YanHuang cells in Dulbecco’s Modified Eagle’s Medium (DMEM, Gibco, CA, USA) for 72 h at a temperature of 38.5 °C in which the 15% FBS, 50 U/mL penicillin, 2 mM L-glutamine and 50 μg/mL streptomycin were contained.

### 4.2. DMS Chemical Probing

Cells were suspended in 2 mL of 1 × DMS reaction buffer in the tube and treated with 15 µL pure DMS. We also prepared control samples by adding 15 µL of nuclease-free water. The experimental and control samples were performed in parallel. The reaction was carried out with periodic swirling at room temperature for 15 min. Then, 0.15 g of DDL was added to the reaction, which was subsequently dissolved through swirling the tube for 2 min. Finally, all of the solution was poured out, and the remaining cells were washed twice with ~50 mL of nuclease-free water.

### 4.3. RNA-Seq

We obtained 3 µg of total RNA for the construction of the library, and quality control was performed using Agilent RNA 6000 nano kit running on Agilent 2100 Bioanalyzer. The RNA passed the quality control with an RNA integrity number >8. We used random hexamers containing Illumina adapters for the reverse transcription, and then the complementary sequence for each fragment was generated. The amplification of fragments was carried out on Illumina, followed by deep sequencing on HiSeq 2000.

### 4.4. Reference Transcriptome Sequence Data

The human reference transcriptome sequence data in FASTA format were obtained from the UCSC Genome Browser Downloads page (https://hgdownload.soe.ucsc.edu/goldenPath/hg38/bigZips/, accessed on 25 June 2021), and the refMrna.fa was chosen as the standard RNA reference sequence. To annotate the reference transcriptome sequence data, we also downloaded the 5′-UTR, 3′-UTR, and CDS (coding sequence) data from the table Browser page in the UCSC Genome Browser website. The genome assembly was set as hg38, while the annotation source was set as GENCODE. To annotate the exact position of 5′-UTR, CDS, and 3′-UTR in each reference transcript, we applied a customized python script, which can map and record the position of the above regions for each transcript based on the download file above.

### 4.5. Transcript Abundance Quantification

Kallisto [30] is an ultra-fast and accurate program that can quantify the transcripts’ abundance from RNA-seq data. In this study, we chose kallisto for transcript abundance quantification (under the default parameters), and the transcripts with TPM (transcripts per million) >1 were retained for the expression level analysis.

### 4.6. Overview of Probing Strategy

**Figure 5:**
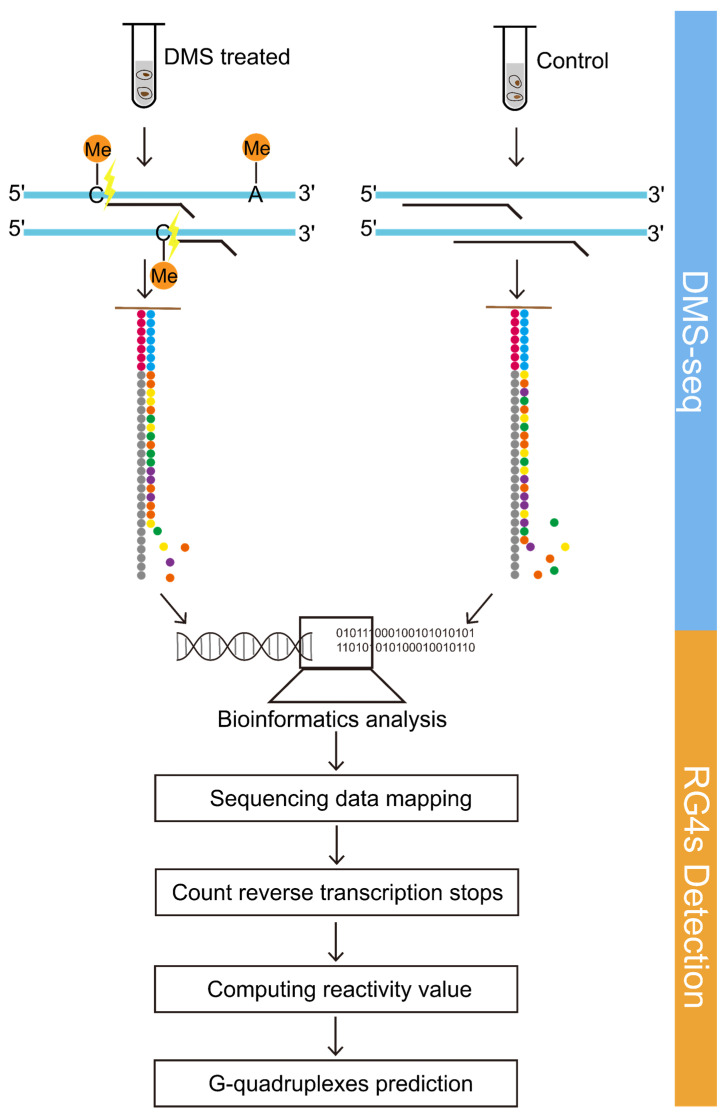
DMS-seq coupling with bioinformatics algorithms detects RNA G-quadruplex structures. The schematic diagram summarizes the brief overview of probing RNA G-quadruplexes. The workflow consists of two parts, including DMS-seq and OG4s detection algorithms.

The workflow of the probing strategy is mainly composed of two parts: DMS-seq and OG4s detection algorithm. We set up the experimental group and the control group, in which the YanHuang cells in the experimental group were treated with DMS, while the control group was not treated with DMS. The computational part was basically derived from the StructureFold [15] software, which was obtained from Galaxy Tool Shed (https://toolshed.g2.bx.psu.edu/repository?repository_id=00fdabcadd09fb14&changeset_revision=7bb98e9296e9, accessed on 25 June 2021). The modified StructureFold [15] software was used to probe the RNA G-quadruplexes under the experimental constraints, which directly reflect the status of nucleotides on each transcript in living cells. Figure 5 illustrates the brief procedure of detecting RNA G-quadruplexes in the human transcriptome based on DMS-seq and bioinformatics algorithms.

### 4.7. Observed RNA G-Quadruplexes Detection Algorithm

We applied and modified the StructureFold [15] software to detect the endogenous RNA G-quadruplexes with experimental constraints. The StructureFold software is composed of four modules: mapping, obtaining RT (reverse transcription) stop counts, calculating reactivity, and predicting RNA G-quadruplexes (Please see ref. [15] for more details). The main purpose of the above modules is to (1) map the sequencing reads to the reference transcriptome; (2) compute the reverse transcription stops for each transcript at a resolution of one nucleotide; (3) calculate the reactivity values by comparing the RT stop files of experimental and control conditions, which can be transformed into the restraints for the prediction; and (4) predict the structure of transcripts based on the RNAfold software with the restraints deriving from reactivity files.

Considering that our sequencing data are paired-end data with a length of 150 nt per read, we then re-customized the mapping module. This module consists of three steps: first, assessing the quality of sequence data using FastQC software; second, cutting the reads through Trimmomatic [31] software based on the report of FastQC, and removing the leading ten bases and the trailing twenty bases on each read; and third, mapping the sequencing reads to the reference transcriptome library by Bowtie2 [32] under the default parameters. The remaining three modules were processed using StructureFold’s built-in programs directly. In the last module, RNAfold [33] (ViennaRNA-2.3.3) was invoked to predict RNA structures with experimental constraints. In summary, we predominantly use the local version of the StructureFold software, but we modified the mapping module to better process our sequencing data.

### 4.8. Calculate RNA G-Quadruplexes Density in 5′-UTRs

Since the median length of the 5′-UTR is about 220 nt, we computed the relative density of RNA G-quadruplexes downstream of the 5′ end using the following two steps:

(1) Aligning all the transcripts to their 5′ end;
(2) For each absolute position i, the relative density $d_{i}$ was defined by the formula below:
  (1) $$d_{i}=\frac{G_{i}}{T_{i}}\times 10^{3}$$
  where $G_{i}$ represents the total number of RNA G-quadruplexes at position i, while $T_{i}$ represents the total number of transcripts longer than i.

The average RNA G-quadruplex density in all of the 5′-UTRs was calculated as follows:

(2) $$Average\;RG4\;density=\frac{l}{L}\times 10^{3}$$

where l represents the total length of the RNA G-quadruplexes in 5′-UTRs, while L represents the total length of all 5′-UTRs.

The relative density of RNA G-quadruplexes upstream of the start codons was calculated in a similar way to the method described above, except that all the transcripts were aligned to their start codons.

### 4.9. Identification of the Putative miRNA Target Sites

The identification of putative miRNA target sites in each transcript was conducted using miRmap [34] and miRanda [35]. These are both open-source software programs, which can provide comprehensive perspectives in predicting putative miRNA target sites. The default parameters and cutoffs were used, except that the energy threshold cutoff was set to −20 kcal/mol in miRanda. The high confidence human miRNA sequences were extracted from the miRBase database (http://www.mirbase.org/, accessed on 25 June 2021). Only the identical prediction targeting sites that appeared in both result datasets generated by the two independent software were utilized as putative miRNA target sites for further analysis.

### 4.10. PAS-Strong Poly(A) Sites Prediction

The POLYAR [36] software was designed to recognize the polyadenylation sites from sequences and has a significant performance in searching for the PAS-strong poly(A) sites. We identified all of the putative PAS-strong poly(A) site positions in 3′-UTR by using POLYAR under the default parameters.

### 4.11. Searching for Unfolded RNA G-Quadruplexes

We developed an R script program to search for all the putative RNA G-quadruplexes in 3′-UTRs, which corresponds to the motif of canonical RNA G-quadruplexes (G_x_N_1−7_G_x_N_1−7_G_x_N_1−7_G_x_, where x ≥ 3 and N can be any base). In order to obtain the unfolded RNA G-quadruplexes, we excluded the observed RNA G-quadruplexes from putative RNA G-quadruplexes, and the remaining putative RNA G-quadruplexes were regarded as UG4s.

### 4.12. Probability of Forming Observed RNA G-Quadruplexes

In this study, we calculated the probability of forming observed RNA G-quadruplexes downstream of the putative PAS-strong poly(A) sites, and the probability is defined as follows:

(3) $$p_{i}=\frac{d_{i}}{d_{i}^{\prime }}\;$$

where $p_{i}$ stands for the probability of forming observed RNA G-quadruplexes at position i in a specific sequence, and $d_{i}$ stands for the density of the observed RNA G-quadruplexes at position i, while $d_{i}^{\prime }$ stands for the density of putative RNA G-quadruplexes at position i.

### 4.13. Enrichment Analysis

The KEGG over-representation testing was performed using the Bioconductor’s clusterProfiler package [37]. The enriched pathways with FDR < 0.05 were retained for the dot plot visualization by using the built-in functions. The pathways and gene interaction networks were visualized through Cytoscape (https://cytoscape.org, accessed on 25 June 2021) software.

The COVID-19 gene set enrichment analysis was conducted in the Enrichr [38] online service. Enrichr [38] is an easy-to-use, open-access website that is special designed for gene set enrichment analysis. We uploaded the gene symbols of the transcripts that contain RNA G-quadruplexes in their 3′-UTRs and obtained a set of significantly enriched (Adjusted *p*-value < 0.05) COVID-19-related gene sets in the Disease/Drugs module of Enrichr.

### 4.14. Simple Random Sampling

The simple random sampling procedure was described as follows,

**Input**: dataset *S*_1_ and *S*_2_; follows *S*_1_ ∩ *S*_2_ = Ø, |*S*_1_| > |*S*_2_|

**Output**: dataset *P*

**while** *i* ≤ 10,000 **do**

*tmp* ← sample(*S*_2_, n = |*S*_1_|)

*P_i_* ← *p* value in wilcox.test (*S*_1_, *tmp*, alternative = greater)

**end**

**return** *P*

## Data Availability

The public data used in this study can be found at the corresponding databases, as is described in the methods section. The raw data reported in this study were deposited in the Genome Sequence Archive for Human (https://bigd.big.ac.cn/gsa-human/, accessed on 25 June 2021) database, which is a part of the Genome Sequence Archive database maintained by the National Genomics Data Center. Data are available under the accession number HRA000832 (https://bigd.big.ac.cn/gsa-human/browse/HRA000832, accessed on 25 June 2021).

## Supplementary Materials

The following are available online at https://www.mdpi.com/article/10.3390/ijms22158012/s1.

## Abbreviations

**Abbreviation**	**Meaning**
CDS	coding sequence
DMS	Dimethyl sulfate
IRES	Internal Ribosome Entry Site
KEGG	Kyoto Encyclopedia of Genes and Genomes
MOIs	multiplicities of infection
NRAS	neuroblastoma RAS viral oncogene homolog
nt	nucleotide
OG4s	observed RNA G-quadruplexes
PAS	Polyadenylation Signal
PD1	Programmed cell Death protein 1
PD-L1	Programmed Death-Ligand 1
RT	reverse transcription
SUD	SARS-unique domain
Th1	T-helper 1
Th2	T-helper 2
Th17	T-helper 17
TPM	transcripts per million
UG4s	Unfolded RNA G-quadruplexes
UTR	untranslated region
