# Supplementary material for: Detecting and Profiling Endogenous RNA G-Quadruplexes in the Human Transcriptome
Source: Int J Mol Sci. 2021 Jul 27;22(15):8012. doi: 10.3390/ijms22158012 (PMC8347560; doi:10.3390/ijms22158012)
Supplement: Supplementary file 1 [file ijms-22-08012-s001.zip › Supplementary Material.pdf]

Supplementary Materials

Detecting and profiling endogenous RNA G-quadruplexes in the human transcriptome

Supplementary Figures

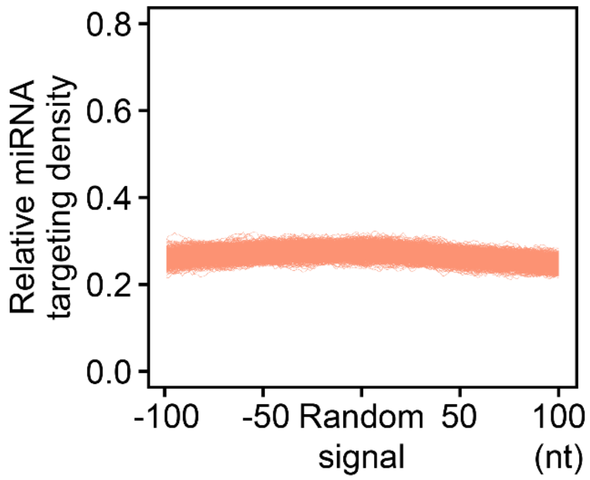

Supplementary Figure S1. The relative miRNA targeting density around random signals. The abscissa represents the distance of 100 nt upstream and downstream of the random signal, while the ordinate represents the relative density. Each line in the plot indicates the result of a randomized trial.

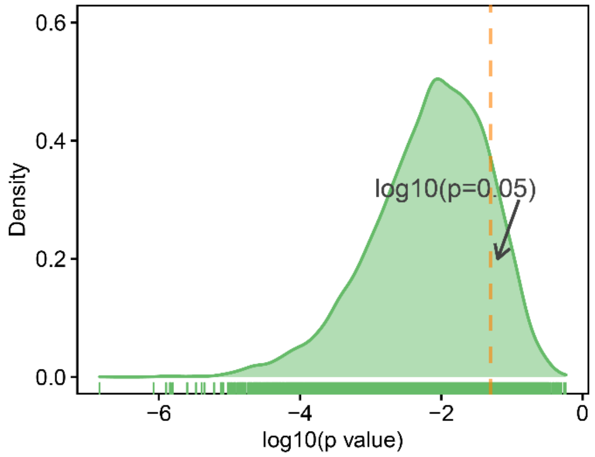

Supplementary Figure S2. Density plot of the p-value obtained from the random sampling rounds. The p-value corresponding to 0.05 was marked as the orange dashed line.

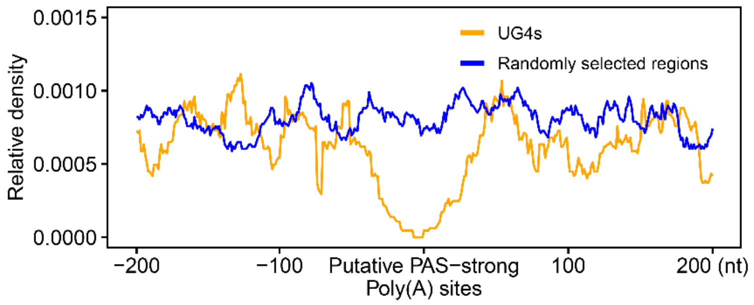

Supplementary Figure S3. The distribution of UG4s at a distance of 200 nt around the putative PAS-strong poly(A) sites. The yellow and blue lines represent the relative density of UG4s and randomly selected regions, respectively.

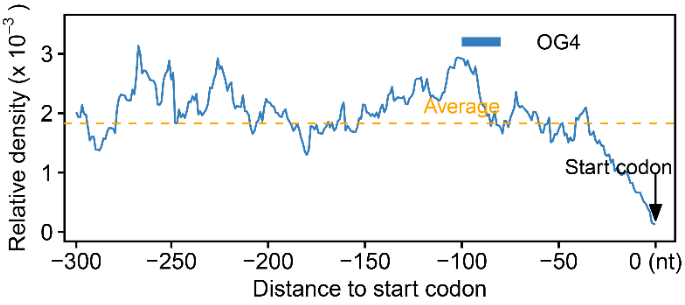

Supplementary Figure S4. The density of the OG4s in 5'-UTRs. The abscissa represents the distance to the start codon, while the ordinate indicates the relative OG4 density value. The dotted line shows the average OG4 density in the whole 5'-UTRs.
